# Indirect Recognition of Predefined Human Activities

**DOI:** 10.3390/s20174829

**Published:** 2020-08-26

**Authors:** Ojan Majidzadeh Gorjani, Antonino Proto, Jan Vanus, Petr Bilik

**Affiliations:** Department of Cybernetics and Biomedical Engineering, Faculty of Electrical Engineering and Computer Science, VSB—Technical University of Ostrava, 70833 Ostrava-Poruba, Czech Republic; antonino.proto@vsb.cz (A.P.); jan.vanus@vsb.cz (J.V.); petr.bilik@vsb.cz (P.B.)

**Keywords:** deep learning, logistic regression, activity recognition, prediction, classification, artificial neural network, smart homes, intelligent buildings

## Abstract

The work investigates the application of artificial neural networks and logistic regression for the recognition of activities performed by room occupants. KNX (Konnex) standard-based devices were selected for smart home automation and data collection. The obtained data from these devices (Humidity, CO_2_, temperature) were used in combination with two wearable gadgets to classify specific activities performed by the room occupant. The obtained classifications can benefit the occupant by monitoring the wellbeing of elderly residents and providing optimal air quality and temperature by utilizing heating, ventilation, and air conditioning control. The obtained results yield accurate classification.

## 1. Introduction

The availability of various affordable and cost-effective technologies for automation drives the rapid increase in smart homes. Such technologies provide the possibility of monitoring and tracking events such as unauthorized entry detection, the status of doors and windows, and presence monitoring. An increase in the number of sensors and integration with the Internet of Things (IoT) within smart homes creates new possibilities for improving the daily life of the residents, such as monitoring the activity and well-being of disabled people or seniors [1].

In recent years the health care and assisted living has gained much attention among researchers. In a case study, Panagopoulos et al. [2] presented a usability assessment of “Heart Around”, an integrated homecare solution incorporating communication functionalities, as well as health monitoring and emergency response features. Loukatos et al. [3] investigated educationally fruitful speech-based methods to assist people with special needs to care for potted plants. Wiljer et al. [4] suggested improving health care by developing an artificial intelligence-enabled healthcare practice. Many of the works in the field of activity recognition are emphasizing fall detections [5,6,7]. Sadreazami et al. [5] proposed using the Standoff Radar and a time series-based method for detecting fall incidents in human daily activities. A time was obtained by summing all the range bins corresponding to the ultra-wideband radar return signals. Ahamed et al. [6] investigated accelerometer-based fall detection, the Feed Forward Neural Network and Long Short-Term Memory based on deep learning networks, applied to detect falls. Dhiraj et al. [7] proposed two vision-based solutions, one using convolutional neural networks in 3D-mode and another using a hybrid approach by combining convolutional neural networks and long short-term memory networks using 360-degree videos for human fall detection.

On a larger scale, Hsuseh et al. [8] adopted deep learning techniques to learn the long-term dependencies from videos for human behavior recognition in a multi-view framework detection. Often, camera-based solutions create concerns regarding security and privacy. Therefore, several works are based on indirect occupancy monitoring. Szczurek et al. [9] investigated occupancy determination based on time series of CO_2_ concentration, temperature and relative humidity. There are works [1] monitoring the daily living activities in smart home care using CO_2_ concentration. Vanus et al. [10] designed an indirect method for human presence monitoring in an intelligent building. Vanus et al. [11] used the IBM SPSS modeler tool and neural networks for CO_2_ prediction within smart home care. Vanus et al. [12] compared neural networks, random trees, and linear regression for the purpose of indirect occupancy recognition in intelligent buildings. This paper proposes to employ an identical KNX-based setup building on the above contributions with a significant difference in expanding the occupancy monitoring to activity recognition.

The indirect recognition of human activity is one of the most highly anticipated research topics. Albert et al. [13] used mobile phones for activity recognition in Parkinson’s patients. Nweke et al. [14] reviewed deep learning algorithms for human activity recognition using mobile and wearable sensor networks. Lara et al. [15] reviewed human activity recognition using wearable sensors and Yousefi et al. [16] reviewed behavior recognition using Wi-Fi channel state information. Minarno et al. [17] compared the performance Logistic Regression and Support Vector Machine to recognize activities such as laying, standing sitting, walking, walking upstairs or downstairs. Kwapisz et al. [18] proposed using logistic regression and multilayer perceptron with data obtained from cell phone accelerometers to recognize similar human activities. In a similar study, Bayat et al. [19] proposed using accelerometer data from smartphones to recognize more complex human activities such as running and dancing. Trost et al. [20] compared results obtained from the hip and wrist-worn accelerometer data for the recognition of seven classes of activities.

This study is aimed at taking the data analysis within smart homes beyond occupancy monitoring and fall detection. Although there are a few available works in the field of activity recognition, this study targets new types of recognizable activities beyond common walking, running, and climbing stairs. The proposed method employs KNX standard-based devices to obtain room air quality data (Humidity, CO_2_, temperature) and combines the obtained data with two wearable gadgets that provide movement-related data. KNX-based devices were selected due to properties such as cost-effectiveness, compatibility and wide availability within locations such as smart homes, office buildings, shopping centers, medical facilities, and industrial locations.

Initially, logistic regression-based models are developed (using IBM SPSS statistic 26) to classify the obtained datasets. Logistic regression is one of the most used methods in the field of activity recognition. Therefore, it provides a good reference for the evaluation of the method using artificial neural networks. Ultimately, the article proposes to use artificial neural networks and the obtained datasets to classify few types of human daily activities such as relaxing, eating, cleaning, exercising using a stationary bike and using a computer. IBM SPSS statistic 26 and IBM SPSS modeler 18 were selected as suitable data analysis platforms to develop required logistic regression and artificial neural network predictive models. In addition to monitoring the wellbeing of elderly residents, the obtained predictions can benefit the occupant by providing optimal air quality and temperature by utilizing heating, ventilation, and air conditioning control. The obtained results yield highly accurate prediction accuracies.

## 2. Materials and Methods

**Figure 1:**
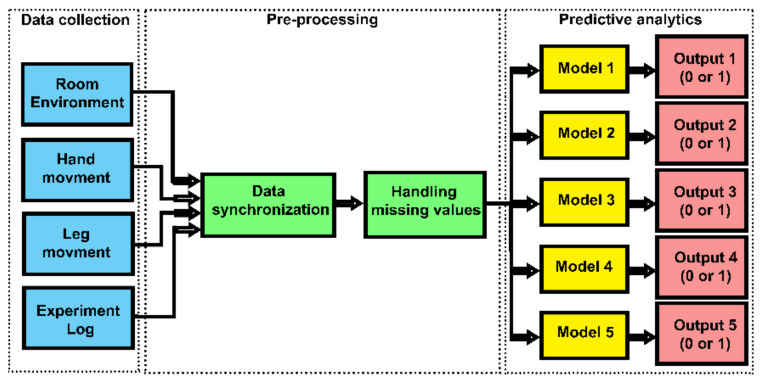
Block diagram of the proposed method using logistic regression.

**Figure 2:**
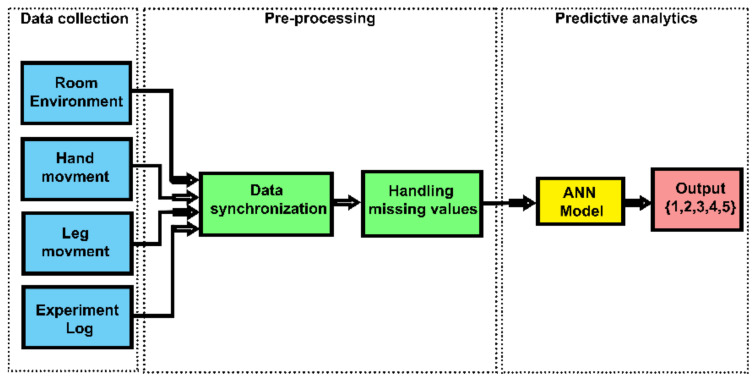
Block diagram of the proposed method using artificial neural networks.

The proposed method contains three main stages of data collection, pre-processing, and predictive analytics (Figure 1). In the first stage, the KNX devices were employed to monitor the air quality of the room in terms of room temperature (C), humidity level (%), CO_2_ Concentration level (ppm). The movements of the room occupant were monitored using two individual wearable gadgets based on the Inertial Measurement Unit (IMU). After data synchronization and dealing with the missing data, predictive analytics were applied. Figure 1 shows the application of logistic regression using IBM SPSS statistics 26. A separate predictive model with binary output was dedicated to each type of activity classes, where 0 represents false and 1 represents true. Since logistical regression is commonly used in this particular field of research, it provides a good benchmark or reference point for the evaluation of the artificial neural network-based method. Figure 2 shows the application of artificial neural networks using IBM SPSS modeler 18. It can be observed that in the second approach a single output was used to determine the outcome of the predictive model.

### 2.1. Data Collection

The data collection was performed in laboratory EB312 at the new Faculty of Electrical Engineering and Computer Science building of the VSB Technical University of Ostrava. The data collection was performed on the 19 July 2019 (08:28:00 to 10:31:00) and 26 July 2019 (08:09:00 to 10:10:00). The activities were performed by a single occupant present in the room. The performed activities were divided into five classes that are described in Table 1. These classes can simulate part of the daily activities performed in a single occupant room.

#### 2.1.1. KNX Technology

A KNX (Konnex) setup was used to monitor the experiment’s room. In general, KNX is an open standard (EN 50090 [21], ISO/IEC 14543 [22]) for commercial and domestic building automation in a variety of locations such as office buildings, shopping centers, medical facilities, and industrial locations. It can be used to control functions such as heating, cooling, ventilation, energy management, and lighting control. The KNX bus system is a decentralized system with multi-master communication. KNX modules are commonly commissioned using the Engineering Tool Software (ETS). In addition to ETS, a .net-based software was developed [12] to ensure the connection of KNX-based devices and IBM cloud storage technology, which enables the communication between IBM Watson IoT platform and KNX smart installation. The measurements of CO_2_ accumulation, indoor temperature, and humidity were performed using the MTN6005-0001 module. The measuring range of this device is listed in Table 2.

#### 2.1.2. Wearable Gadgets

**Figure 3:**
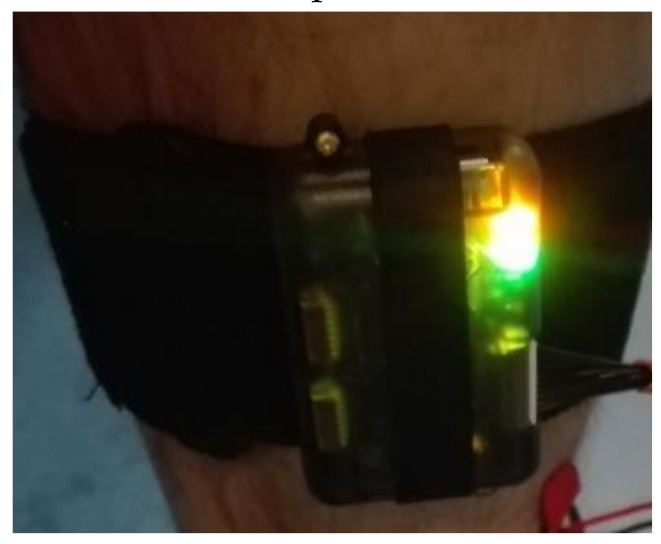
Inertial Measurement Unit (IMU) worn on a leg.

Two wearable gadgets were used to monitor the experimenter’s movements [23,24]. One was worn on the right hand and the other on the right leg (Figure 3). The wearable gadgets were based on the new generation of the Inertial Measurement Unit (IMU), developed by x-io Technologies, UK. The IMU is a compact data acquisition platform that combines diverse onboard sensors (as displayed in Table 3), and it is largely used for the evaluation of gait variability [24,25]. As regards this study, it comprises an 8-channel analog input, and an SD-card to store the data. The analog input of the IMU is equipped with a 10-bit AD-converter that allows us to acquire and convert the signals from a variety of modules. Table 3 shows the measured parameters and their units.

### 2.2. Pre-Processing

The wearable gadgets are using an approximated data collection rate of 30 to 60 samples per second and the KNX-based data collection rate is between 1 to 10 samples per minute. This large difference creates a database synchronization problem. Therefore, the data collected from KNX devices had been expanded to match the fast rates of the wearable gadget. A .Net-based script was used to perform data synchronization. Missing data could result in algorithm failure or decrease the accuracy of the analysis. Therefore, IBM SPSS software tool automatically removes all of the records with missing data from the analysis. Using the IBM SPSS software tool time-related variables were removed and correct variable types were assigned to each parameter (continuous and binary).

### 2.3. Predictive Analytics

Predictive modeling is the general concept of building a model that uses big data to develop models capable of making reliable predictions. In general, these models are based on variables (also known as predictors) that are most likely to influence the outcome [26]. Predictive models are widely applied in various applications such as weather forecasting [27,28,29], Bayesian spam filters [30,31,32,33], business [34,35,36,37], and fraud detection [38,39,40]. Predictive models typically include a machine learning algorithm that learns certain properties from a training dataset. The learning process can be applied using supervised learning [41,42], unsupervised learning [42], semi-supervised learning [43], active learning. In the purposed method, supervised learning was employed by presenting a set of solved (labeled) examples to the model for training. Once the model is established, a pattern between the predictors and the outcome could solve similar predictions on its own. 

#### 2.3.1. Logistic Regression

**Figure 4:**
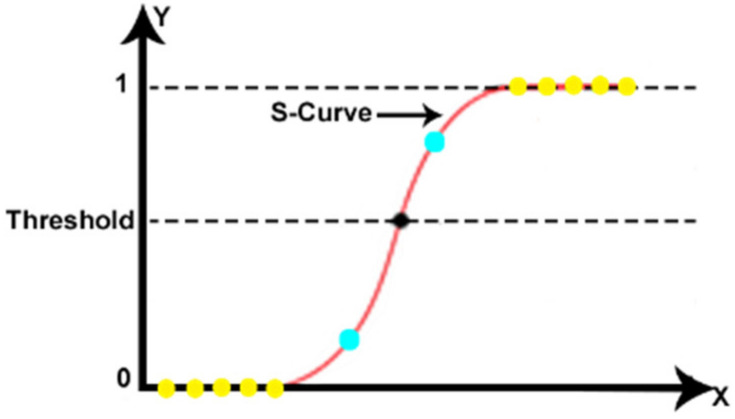
Example of the logistic function.

Regression is one the oldest and often used algorithms in machine learning with a supervised learning strategy [44,45]. Linear Regression and Logistic Regression are the two famous types of regression. In general, Linear Regression is used for solving Regression problems whereas Logistic Regression is used for solving the Classification problems such as predicting the categorical dependent variable with the help of independent variables or where the probabilities between two classes are required [45]. Logistic regression is used in various fields, including machine learning, most medical fields, and social sciences [46,47,48,49,50]. The weighted sum of inputs passes through the logistic function Equation (1) that can map values in between 0 and 1. The logistic function is a sigmoid function [51] and the curve obtained is called a sigmoid curve or S-curve (Figure 4).

The output of the binary logistic regression model can be only binary (either 0 or 1). Outputs with more than two values are modeled by multinomial logistic regression and if the multiple categories are ordered, by ordinal logistic regression. The logistic regression is not a classifier by itself, it simply provides a probability of output in terms of input. However, it can be used to make a classifier, for instance by choosing a cutoff value and classifying inputs with probability greater than the cutoff as 1 and below the cutoff as 0; this is a common way to make a binary classifier. The general equation of logistic regression is provided by Equation (2).

(1) $$f\left(x\right)\;=\;\frac{1}{1\;+\;e^{-k\left(x-x_{0}\right)}}$$

(2) $$y\;=\;\frac{1}{1\;+\;e^{-(\beta_{0}+\beta_{1}x_{1}+\beta_{2}x_{2}+\beta_{3}x_{3}+\cdots +\beta_{n}x_{n})}}$$

Regression models can be created using multiple algorithms, these algorithms specify how independent variables are entered into the model [52,53,54,55,56]. The common algorisms are Enter (Regression) [56,57], Stepwise [58], Backward Elimination [59] and Forward Selection [60,61]. The Hosmer–Lemeshow test and Omnibus test are some of the most common statistical tests used to examine the goodness of fit for logistic regression. It compares the observed event rates and expected event rates in subgroups of the model population. The test mainly identifies subgroups as the deciles of fitted risk values. Well calibrated models are the models with similar expected and observed event rates in their subgroups. The expected probability of success is given by the equation for the logistic regression model. In general, the Hosmer–Lemeshow test is useful to determine if the lack of fit (poor prediction) is significant but it does not properly take overfitting into account. The omnibus test is a likelihood-ratio chi-square test of the current model versus the null (in this case, the intercept) model. Generally, the significance value of less than 0.05 indicates that the current model outperforms the null model. The odds ratio is often used to quantify the strength of the association between two events. In logistic regression, the odds ratio shows the amount of increase in the output variable with every unit increase in a specific input variable. The odds ratio for a continuous independent variable can be defined as Equation (3). This exponential relationship provides an interpretation for $\beta_{1}$ where the odds is multiplied by $e^{\beta_{1}}$ for every 1-unit increase in x [62]. If a, b, c and d can are cells in a 2 × 2 contingency table then formula 4 describes odds ratio for a binary independent variable.

(3) $$odds\;ratio\left(OR\right)\;=\;\frac{p\;\left(x\;+\;1\right)}{p\left(x\right)}\;=\;\frac{e^{\beta_{0}+\beta_{1}\left(x+1\right)}}{e^{\beta_{0}+\beta_{1}\left(x\right)}}\;=\;e^{\beta_{1}}$$

(4) $$odds\;ratio\left(OR\right)\;=\;\frac{ad}{bc}$$

#### 2.3.2. Artificial Neural Network

Due to their power flexibility and ease of use, artificial neural networks are widely used [63,64,65,66,67,68,69]. Artificial neural networks obtain their knowledge from the learning process and then use interneuron connection strengths (known as synaptic weights) to store the obtained knowledge [70,71]. One of the most used classes of artificial neural networks is a multilayer perceptron which is a feedforward neural network that belongs to deep learning. Deep learning utilizes a hierarchical level of artificial neural networks to carry out the process of machine learning. Unlike traditional programs, the hierarchical function of deep learning systems enables machines to process data with a nonlinear approach. The multilayer perceptron utilizes backpropagation for training [72,73,74]. Due to its multiple layers and nonlinear activation, a multilayer perceptron can distinguish data that are not linearly separable [75].

**Figure 5:**
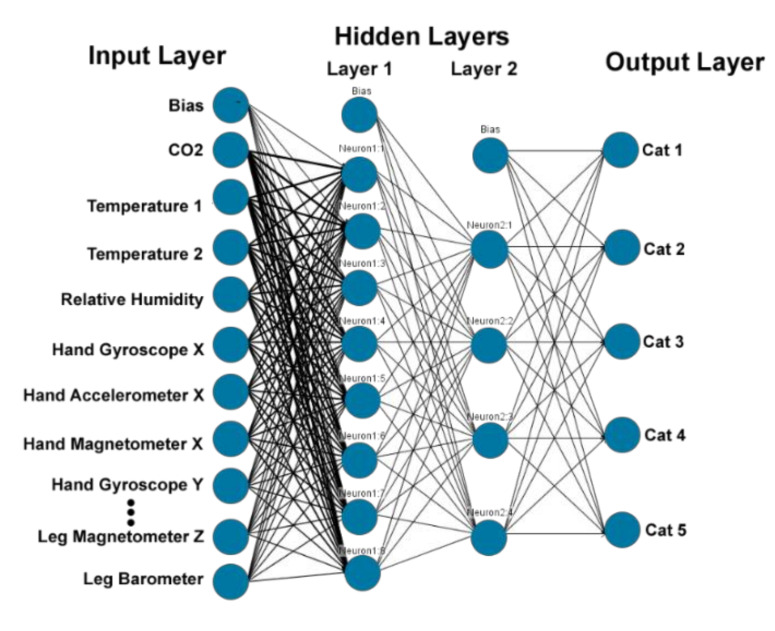
Example of the developed multilayer perceptron artificial neural network model with 24 neurons input layer, eight neurons in the first hidden layer, four neurons in the second hidden layer, five neurons output layer.

In deep learning, in addition to input and output, layers of the neural network contain multiple hidden layers and each can contain multiple neurons. The first layer of the neural network processes a raw data input like the amount of the transaction and passes it on to the next layer as output. The second layer processes the previous layer’s information by including additional information. This continues across all levels of the neural network. Each layer of its neural network builds on its previous layer. The multilayer perceptron artificial neural network with two hidden layers was chosen as a suitable deep learning method for this article (Figure 5).

The multilayer perceptron artificial neural network was implemented in the IBM SPSS Modeler 18 software. The IBM SPSS modeler algorithm guide mathematically describes its multilayer perceptron model as followings [76]:

**Input layer:**$j_{0}\;=\;p$ units, $a_{0:j},\ldots ,a_{0:j0}$, with $a_{0:j}\;=\;xj$, where j is the number of neurons in the layer and X is the input.

**ith hidden layer:**$j_{i}$ units, $a_{i:1},\ldots ,a_{i:j_{i}}$, with $a_{1:k}\;=\;\gamma_{i}\left(C_{i:k}\right)$ and $C_{i:k}\;=\;\sum_{j=0}^{j_{i-1}}\omega_{I:j1},{\;k}^{a_{i-1:j}}$, where $a_{i-1:0}\;=\;1$, $\gamma_{i}$ is the activation function for the layer I, and $\omega_{I:j1}$ is weight leading from layer i−1. At this layer, the model uses hyperbolic tangent as an activation function provided by $\gamma_{\left(C\right)}\;=\;\tanh\left(c\right)\frac{e^{c}\;-\;e^{-c}}{e^{c}\;+\;e{-}^{c}}$.

**Output layer:**$j_{I}\;=\;R$ units, $a_{I:1},\ldots ,a_{I:J_{I}}$, with $a_{I:k}\;=\;\gamma_{I}\left(C_{I:k}\right)$ and $C_{I:k}\;=\;\sum_{J=0}^{J_{1}}\omega_{I:j},\;k^{a_{i-1:j}}$, where $a_{i-1:0}=1$. The SoftMax function ($\gamma_{\left(C_{k}\right)}\;=\;\frac{e^{c_{k}}}{\sum_{j\in \Gamma_{h}}e^{c_{j}}}$) is used as an activation function.

To evaluate the performance of predictive modeled three methods of splitting, partitioning, and scoring is commonly used. In the partitioning method, the datasets are randomly divided into training, testing, and validation partitions where models are trained, tested and evaluated using different segments of the dataset. Partitioning is mostly recommended for very large datasets. The scoring method uses entirely different datasets for training and evaluation. One dataset is solely used for training and a separate dataset for evaluation. Therefore, it provides a better indication of the real accuracy of the models.

## 3. Implementation and Results

This section discusses the implementation and results of the classifications performed by logistic regression and artificial neural networks (multilayer perceptron). The logistic regression is a commonly used classification method in the field of activity recognition. Therefore, it can provide a good comparison point for the main purposed method using artificial neural networks.

### 3.1. Linear Regression

The obtained data from measurements performed on the 19 July 2019 (dataset A) and 26 July 2019 (dataset B) were analyzed using IBM SPSS Statistics 26 software tool. Since IBM SPSS Modeler 18 does not natively include logistic regression, IBM SPSS Statistics 26 software was used to perform the logistic regression analysis. In the first stage, the datasets A and B were individually classified. The logistical regression models were developed using enter configuration, classification cutoff of “0.5” and a maximum of 20 iterations.

The goodness of fit describes how well a statistical model fits a set of observations. Hosmer and Lemeshow and omnibus tests were used to determine the goodness of fit. For a good fit, the Hosmer & Lemeshow test significance value should be more than 0.05 and omnibus should have a significance value less than 0.05. These conditions were satisfied with large margins across all models. Table 4 and Table 5 show the accuracy of classification for data obtained from measurements of dataset A (total of 296,188 records) and dataset B (total of 290,174 records). The result shows that all models obtained classification accuracy above 91.2%. In analysis performs on the measurement interval of dataset A (Table 4), the activity Class 3 shows almost complete accuracy (only two wrong predictions in 296,188 records) and activity Class 4 shows the lowest accuracy (97.4%).

Similar characteristics can be observed from the dataset B (Table 5) result where Class 3 yields the highest accuracy (99.9%) and Class 4 the lowest (91.2%). Table 1 indicates that Class 4 is dedicated to cleaning activities such as wiping tables and vacuum cleaning. Therefore, the lower accuracy could be the direct result of less consistent movement during this activity class. Using a stationary bicycle (Class 5 activity) is a high energy activity, and on the contrary, relaxing with minimal movements (Class 1) is a low energy activity. Regardless of energy levels, both of these activities provide consistent movements that directly translate to a more recognizable pattern within data. This can be easily observed within the classification results (99.3% and 98.9% for Class 5 and 98.9 and 99.5% for Class 1). Summing up the classification accuracy resulted in 97.8% of correctly classified records.

Table 6 shows the odds ratio of different parameters in developed models. The odds ratio shows the amount of increase in the output variable with every unit increase in a specific input variable. Simply, the output variable is more associated with changes in parameters with a larger absolute value of the odds ratio. Table 6 shows consistent odds ratios for gyroscope and magnetometer (both devices and across all three axes), CO_2_ for all models. Therefore, it affects all models with a similar significance. 

By comparing each model with its alternative interval, it can be observed that except for Model 5, the temperature has a similar range on both datasets. However, models 1, 2, and 4 share the very high odds ratio and model 3 shows a null effect. Table 6 also shows that this null effect is also shared with the models based on the dataset A. It is also apparent that KNX-based data do not influence the recognition of Class 3 activity. On the other hand, the accelerometer y-axis (both devices) share similar large odds Table 6 shows the odds ratio of different parameters in developed models. The odds ratio shows the amount of increase in the output variable with every unit increase in a specific input variable. Simply, the output variable is more associated with changes in parameters with a larger absolute value of the odds ratio. Table 6 shows consistent odds ratios for gyroscope and magnetometer (both devices and across all three axes), CO_2_ for all models. Therefore, it affects all models with a similar significance.

By comparing each model with its alternative interval, it can be observed that except for Model 5, the temperature has a similar range on both datasets. However, models 1, 2, and 4 share the very high odds ratio and model 3 shows a null effect. Table 6 also shows that this null effect is also shared with the models based on the dataset A. It is also apparent that KNX-based data do not influence the recognition of Class 3 activity. On the other hand, the accelerometer y-axis (both devices) share similar large odds ratio across both models, and in the case of exercising using a stationary bicycle (Class 5 activity), this large effect can be observed on the X-axis of the leg accelerometer.

With few exceptions, the odds ratio of both datasets remains within a similar range, this indicates the consistency of the analysis. Overall, it can be observed that activity Class 1 is mainly affected by temperature and the Activity Classes 2, 4, 5 are mostly affected by temperature and are accelerometer-based. The obtained conclusions from the odds ratio were verified by regression weights, the test of significance, and Wald statistic. In the last stage of the analysis, the developed models were further evaluated by alternative datasets (scoring), resulting in a significant drop in the prediction accuracy (up to 50% decrease). This indicated is an indication of overfitting. Although Hosmer and Lemeshow and omnibus tests are a good indication for the goodness of fit, they do not detect overfitting.

Across both models, and in the case of exercising using a stationary bicycle (Class 5 activity), this large effect can be observed on the X-axis of the leg accelerometer.

With few exceptions, the odds ratio of both datasets remains within a similar range, this indicates the consistency of the analysis. Overall, it can be observed that activity Class 1 is mainly affected by temperature and the Activity Classes 2, 4, 5 are mostly affected by temperature and are accelerometer-based. The obtained conclusions from the odds ratio were verified by regression weights, the test of significance, and Wald statistic. In the last stage of the analysis, the developed models were further evaluated by alternative datasets (scoring), resulting in a significant drop in the prediction accuracy (up to 50% decrease). This indicated is an indication of overfitting. Although Hosmer and Lemeshow and omnibus tests are a good indication for the goodness of fit, they do not detect overfitting.

### 3.2. Artificical Neural Network

**Figure 6:**
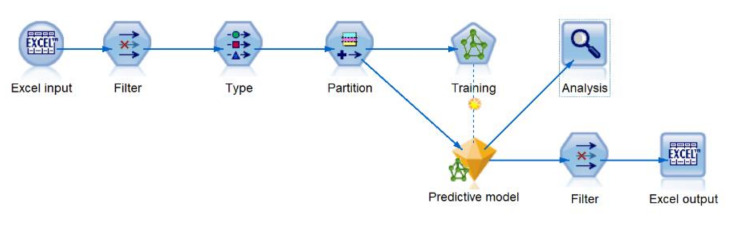
Training and validation stream developed in IBM SPPSS modeler.

The IBM SPSS Modeler 18 software tool was used to create the multilayer perceptron artificial neural networks. Figure 6 displays the data stream developed to train, test, and validate predictive models. In the first stage, the datasets A and B were imported to the data stream. To maintain the integrity of the analysis, the excel node was configured to exclude (delete) the records that contain missing values. The filter and type nodes were utilized to select relevant input data, assign correct variable types (continuous, categorical, etc.), and predefining inputs and the outputs.

The data stream uses a partitioning node with a partitioning ratio of 40% training, 30% testing, and 30% validation. The random seed of “229176228” was set automatically by the partitions node. The stream continues with the artificial neural networks modeling node (training) which generates the predictive model (displayed as nugget gem). In artificial neural network training, the stopping rules settings, the use minimum accuracy, and the customized number of maximum training cycles were disabled, and the maximum training time per component model was set to 15 minutes. Additionally, overfit protection was set to 30%. The predictive model (nugget gem) can be connected to additional nodes to export its predictions to Excel files (using excel node) or analyze them using built-in functions. Table 7 and Table 8 show the results of the training stage.

**Figure 7:**
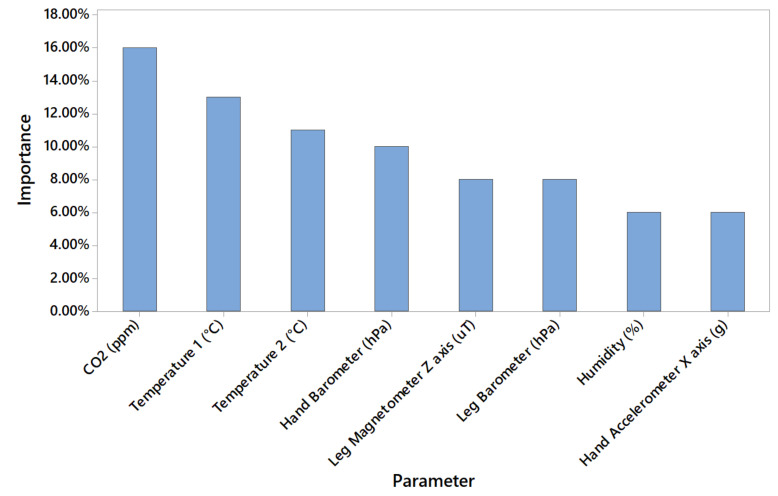
Predictors importance for model 3 trained with dataset A (19 July 2019 interval).

Table 7 and Table 8 show the accuracy of validation partitions for each class in addition to the overall accuracy which is based on all partitions and parameters. Table 7 is based on training results using dataset A and Table 8 is based on training results using dataset B. Similar results can be observed in both training intervals. Model number 3 shows the highest and model 11 the lowest accuracy levels. It can also be observed that the accuracy of the models 1 to 6 are above 99.50% while models 7 to 11 show poor results. Further investigations showed that the models with lower number neurons are mainly based on KNX-based parameters (CO_2_, temperature, humidity) which change at a slower rate. An example of the predictor’s importance for model 3 trained with dataset A is provided in Figure 7.

**Figure 8:**
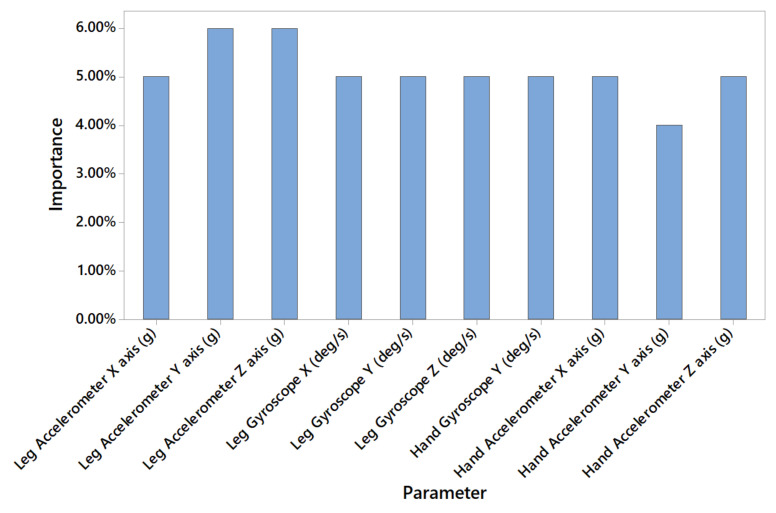
Predictors importance for model 11 trained with dataset A (19 July 2019 interval).

Meanwhile, the models with higher neuron count are mainly based on wearable gadget data (accelerometer sensors and gyroscopes) which change at a much faster pace. An example of the importance of predictors for model 11 trained with a dataset A is provided in Figure 8. Additionally, Figure 8 shows that predictors important are more balanced in comparison with Figure 7.

**Figure 9:**
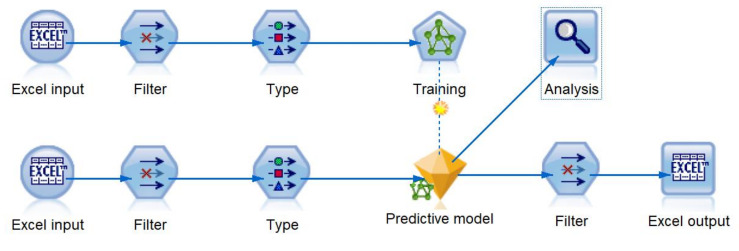
Scoring stream built in IBM SPPSS modeler.

As mentioned earlier, the partitioning is a commonly used method, as an evaluation method. However, soring is commonly applied to obtain a better understanding and estimate of the real performance of the models. In scoring, two different datasets are introduced. One dataset is solely used for training and a separate dataset for evaluation, i.e., the predictive model never sees the input data used for evaluation. Figure 9 shows the scoring diagram implemented in the IBM SPSS modeler 18. The top row of nodes is used for the training data and the bottom for the evaluation data.

Table 9 and Table 10 represent the results obtained from the scoring stage where Table 9 shows the models trained with dataset A evaluated with interval dataset B and Table 10 shows the models trained with dataset B evaluated with dataset A. In Table 9, For models 1 to 6 and activities Classes 1, 3, and 5, we can observe consistent and acceptable results. The highest accuracy for activity Class 2 was achieved by model 1 (94.26%). Meanwhile, model 2 showed the highest accuracy (64%) for activity Class 4. In general, the accuracy of Class 2 and Class 4 are inconsistent. Table 10 shows that models 2 and 3 show better overall accuracy. In particular, model 2 has the highest accuracy for activity Classes 1, 2, 3, and 5. 

## 4. Discussion

This article proposes recognizing human activities in a single occupant room using room air quality data (Humidity, CO_2_, temperature) in combination with movement-based data (accelerometer, gyroscope, magnetometer). The measured data were classified using logistic regression and multilayer perceptron artificial neural networks. Logistical regression is commonly used in this particular field of research. Therefore, it can provide a good reference point for the evaluation of the artificial neural network-based method. The Hosmer and Lemeshow test and omnibus test showed a good fit for the models. The result showed an average classification accuracy of 97.8% and a minimum average accuracy of 91.2%. The accuracy of models based on dataset A which ranged between 97.4% to 100%, and for dataset B, the accuracy ranged between 91.2% and 99.9%. In both datasets, Class 3 yields the highest accuracy and Class 4 the lowest. The main contributor to the reduced classification accuracy was identified as less consistent movements during cleaning activity. On the contrary, relaxing (Class 1) and using a stationary bike (Class 5) are relevantly consistent activities (with regards to movement patterns), hence the higher accuracy was observed. To develop a better understanding of the results, the obtained models were examined in terms of the odds ratio, regression weights, test of significance, and Wald statistic. With some exceptions, the odds ratio of both datasets remained within a similar range which is a good indication of consistency within the analysis result. Further investigations showed that that activity Class 1 is mainly affected by temperature and the activity Classes 2, 4, 5 are mostly affected by temperature and accelerometer-based data.

Once the logistic regression set an accuracy reference point, the multilayer perceptron artificial neural networks were implemented in the IBM SPSS Modeler 18 software. Initially, the models were developed and evaluated using a partitioning method resulting in relevantly similar validation results for both datasets. Model 3 held the highest accuracy, and model 11 the lowest, the models 1 to 6 were all above 99.50% while models 7 to 11 showed less impressive results. Deeper investigations showed that the models with lower number neuron count (models 1 to 6) are mainly influenced by room measurement data (CO_2_, temperature, humidity). Meanwhile, the models with a higher number of neurons were mainly based on wearable gadget data (accelerometer sensors and gyroscopes) which change at a much faster pace. In the latter case, the important predictors were more balanced and spread. The validation results of multilayer perceptron models 1 to 6 surpassed the accuracy logistic regression by staying above 98.92% in all classes where the logistical regression score average of 97.8%.

The superiority of the artificial neural networks became significantly more apparent by performing model scoring where a significant decrease in accuracy was observed with logistic regression models. When it comes to scoring, a decrease in prediction accuracy is always expected. Increasing the size of the training datasets closes the gap between the validation and scoring accuracy. In the case of logistic regression, the gap was significant enough to conclude that the models are overfitting. In the case of artificial neural networks, the results were more consistent and acceptable for Activity Classes 1, 2, 3, and 5. Similar to the previous cases, Class 4 performed poorly due to inconsistencies within movements during this activity class.

Overall, the obtained results showed that the proposed method provides a promising outcome. Although there are many contributions in the field of human activity recognition, this study holds its novelty in terms of methodology, measurement techniques, and predefined classes. In terms of accuracy of activity recognition, this study is on par or surpasses most of the similar previous works. In terms of predefined activities, this article studied new classes of activities where similar works are mainly focused on stairs climbing, running, walking, and fall detection. The predefined activities in this article do not represent all possible daily activities performed by humans. However, the highly accurate results obtained in this article show a promising path for expansion. In future works, the study will expand in terms of number recognizable activates in addition to the possibilities of recognition within multiple occupant rooms.

## 5. Conclusions

This article proposed recognizing human activities in a single occupant room using room air quality data (Humidity, CO_2_, temperature) in combination with movement-based data (accelerometer, gyroscope, magnetometer). The measured data were used to recognize five predefined human activity classes such as relaxing, using a computer, eating, cleaning and exercising. The classification was performed using logistic regression and artificial neural networks (multilayer perceptron) where logistic regression was used as a reference for the evaluation of the main purposed method that is using artificial neural networks. In comparison with similar studies, this study holds its novelty in terms of methodology, measurement techniques and predefined classes.

The neural network showed more consistent and acceptable results. Although the obtained classification accuracy varied depending on the type of performed activity, the overall results were highly accurate. In general, a promising outcome and highly accurate results were obtained in this analysis which shows a promising path for expansion in the number of recognizable activity classes and possibilities of recognition within multiple occupant rooms.

## Figures and Tables

**Table 1:** Description of activity categories.

Activity Class	Description
Class 1	Relaxing with minimal movements
Class 2	using the computer for checking emails and web surfing
Class 3	Preparing tea and sandwich—eating breakfast
Class 4	Cleaning the room by wiping the Tables and vacuum cleaning
Class 5	Exercising using stationary bicycle

**Table 2:** List of measured parameters and their unit.

Sensor	Unit	Range
CO_2_	ppm	300 to 9999
Temperature 1		0 to +40
Relative humidity sensor	%	20 to 100

**Table 3:** List of measured parameters using wearable gadgets.

Parameter	Unit
Gyroscope X, Y, Z	deg/s
Accelerometer X, Y, Z	g
Magnetometer X, Y, Z	μT
Barometer	hPa

**Table 4:** Classification table using dataset A (19 July 2019 interval).

Class	Observed	Predicted		Percentage Correct	Overall Accuracy
		0	1		
Class 1	0	273,204	2758	99.9%	98.9%
	1	422	19,804	97.9%	
Class 2	0	213,908	3764	98.3%	97.4%
	1	3877	74,639	95.1%	
Class 3	0	220,320	0	100.0%	100.0%
	1	2	75,866	100.0%	
Class 4	0	243,244	5327	97.9%	95.4%
	1	8209	39,408	82.8%	
Class 5	0	223,644	1096	99.5%	99.3%
	1	869	70,579	98.8%	

**Table 5:** Classification table using dataset B (26 July 2019 interval).

Activity	Observed	Predicted		Percentage Correct	Overall Accuracy
		0	1		
Class 1	0	270,378	867	99.7%	99.5%
	1	640	18,829	96.7%	
Class 2	0	213,182	3791	98.3%	97.0%
	1	5068	68,673	93.1%	
Class 3	0	216,883	95	100%	99.9%
	1	51	73,685	99.9%	
Class 4	0	235,594	9914	96.0%	91.2%
	1	15,720	29,486	65.2%	
Class 5	0	212,940	1653	99.2%	98.9%
	1	1535	74,586	98.05	

**Table 6:** Odds ratio.

Activity Class			Dataset A					Dataset B				
			1	2	3	4	5	1	2	3	4	5
**KNX**	Humidity		0.02	0.00	0.00	0.00	0.00	1.59 × 10^3^	1.03 × 10^2^	0.00	0.37	1.45 × 10^8^
	Temperature		2.29 × 10^62^	1.40 × 10^25^	0.00	3.29 × 10^3^	6.66 × 10^36^	1.55 × 10^45^	5.29 × 10^28^	0.00	4.45 × 10^3^	1.03 × 10^11^
	CO_2_		0.94	0.89	0.31	0.86	0.71	0.12	0.99	0.00	0.96	1.05
**Leg**	Gyroscope	x	1.00	0.99	0.94	1.00	1.00	0.99	1.00	1.05	1.00	0.99
		y	0.99	0.99	1.05	1.00	1.02	0.99	0.99	0.92	1.00	1.01
		z	1.00	0.99	0.96	1.00	0.99	0.99	0.99	1.02	1.00	1.01
	Accelerometer	x	4.20	0.55	7.44	1.57	0.27	4.98	0.79	0.00	1.35	0.34
		y	0.43	5.32	5.68 × 10^4^	2.16	0.37	2.52	1.62	5.75 × 10^5^	1.23	0.28
		z	0.09	0.18	0.00	3.17	4.97 × 10	0.02	0.34	0.01	1.61	2.14
	Magnetometer	x	0.99	0.99	1.65	1.01	0.99	0.99	0.95	1.17	1.01	1.04
		y	1.05	1.02	0.67	1.02	0.95	1.08	1.00	0.90	1.03	0.89
		z	1.02	0.89	0.37	1.06	1.17	0.92	0.93	0.92	1.02	1.24
**Hand**	Gyroscope	x	1.00	1.00	1.03	1.00	1.00	1.00	1.00	1.02	1.00	1.00
		y	1.00	1.00	1.07	1.00	1.00	1.01	1.00	1.03	1.00	1.00
		z	0.99	0.99	1.09	1.00	1.00	0.99	1.00	1.03	1.00	1.00
	Accelerometer	x	0.06	0.67	0.35	0.08	8.07 × 10	3.53	8.58	3.09	0.07	1.79 × 10
		y	3.30	1.50 × 10	1.67 × 10	0.14	0.14	0.01	0.19	8.01 × 10^2^	0.68	1.85
		z	9.42	0.04	0.00	2.78 × 10	1.13	0.87	3.99 × 10	0.08	0.05	4.28
	Magnetometer	x	0.97	0.97	0.44	0.97	1.08	1.01	1.05	1.98	1.00	1.05
		y	1.08	1.067	0.235	0.967	0.939	1.024	0.969	0.662	0.987	1.01
		z	1.00	0.96	0.86	1.01	1.08	1.11	1.07	1.66	0.99	0.95

**Table 7:** Validation result of training using dataset A (19 July 2019 interval).

Model	Number of Neurons		Overall Accuracy	Accuracy				
	Hidden Layer 1	Hidden Layer 2		1	2	3	4	5
1	8	4	99.80%	99.05%	99.99%	99.85%	99.95%	99.96%
2	16	8	99.90%	99.79%	99.99%	99.88%	99.96%	99.97%
3	16	32	99.99%	99.81%	99.99%	99.91%	99.98%	99.98%
4	64	32	99.90%	99.76%	99.97%	99.91%	99.93%	99.94%
5	64	128	99.50%	99.22%	99.63%	99.67%	99.38%	99.26%
6	128	64	99.60%	98.92%	99.83%	99.68%	99.74%	99.64%
7	128	256	96.40%	96.02%	96.59%	96.89%	97.12%	94.97%
8	256	128	98.50%	98.44%	98.93%	98.87%	98.85%	97.29%
9	256	512	76.40%	76.48%	77.04%	78.08%	76.81%	73.26%
10	512	256	82.80%	83.98%	82.30%	83.84%	82.94%	80.06%
11	512	512	73.10%	74.52%	71.98%	73.63%	71.9%	73.4%

**Table 8:** Validation result of training using dataset B (26 July 2019 interval).

Model	Number of Neurons		Overall Accuracy	Accuracy of Validation Partition				
	Hidden Layer 1	Hidden Layer 2		1	2	3	4	5
1	8	4	99.70	99.20%	99.97%	99.78%	99.71%	99.73%
2	16	8	99.80	99.81%	99.90%	99.91%	99.71%	99.63%
3	16	32	99.90	99.70%	99.97%	99.92%	99.94%	99.95%
4	64	32	99.80	99.66%	99.89%	99.91%	99.76%	99.67%
5	64	128	99.50	99.56%	99.62%	99.79%	99.43%	99.02%
6	128	64	99.50	99.43%	99.78%	99.7%	99.45%	99.27%
7	128	256	96.70	97.50%	96.55%	96.74%	97.21%	95.38%
8	256	128	98.50	98.53%	98.69%	98.95%	99.07%	97.04%
9	256	512	76.40	77.13%	81.02%	73.32%	75.54%	75.47%
10	512	256	83.30	84.31%	85.28%	80.45%	84.27%	82.52%
11	512	512	74.00	73.75%	75.70%	70.20%	75.69%	74.00%

**Table 9:** Scoring result of training dataset A (19 July 2019 interval) and evaluation dataset B (26 July 2019 interval).

Model	Neurons In Hidden Layers		Accuracy of Each Activity Class				
	Layer 1	Layer 2	1	2	3	4	5
1	8	4	93.30%	94.26%	73.82%	47.23%	84.45%
2	16	8	93.30%	25.36%	73.82%	64.37%	84.45%
3	16	32	93.30%	25.36%	73.82%	25.37%	84.45%
4	64	32	93.30%	74.64%	73.82%	25.53%	84.42%
5	64	128	93.30%	77.17%	75.37%	25.47%	84.44%
6	128	64	93.30%	71.41%	73.02%	25.37%	84.45%
7	128	256	6.81%	46.69%	73.64%	25.58%	30.11%
8	256	128	93.30%	82.13%	41.33%	25.39%	84.45%
9	256	512	89.21%	40.60%	50.34%	34.60%	40.89%
10	512	256	92.94%	25.42%	68.58%	41.13%	84.26%
11	512	512	30.37%	30.83%	74.14%	35.67%	63.93%

**Table 10:** Scoring result of training dataset B (26 July 2019 interval) and evaluation dataset A (19 July 2019 interval).

Model	Neurons In Hidden Layers		Accuracy of Each Activity Class				
	1	2	1	2	3	4	5
1	8	4	50.83%	88.64%	75.88%	63.5%	83.92%
2	16	8	93.17%	98.51%	75.88%	67.11%	96.2%
3	16	32	81.21%	88.59%	75.88%	55.81%	76.11%
4	64	32	27.6%	88.79%	75.88%	66.03%	68.66%
5	64	128	82.01%	82.30%	75.88%	77.77%	44.14%
6	128	64	77.94%	92.65%	75.88%	73.02%	50.21%
7	128	256	17.71%	49.58%	75.71%	26.85%	44.48%
8	256	128	22.60%	85.72%	75.87%	64.71%	30.62%
9	256	512	40.22%	44.92%	50.45%	49.82%	63.95%
10	512	256	33.51%	13.1%	77.95%	52.3%	29.81%
11	512	512	70.2%	78.37%	48.53%	56.77%	33.39%
